# Mesenchymal stromal/stem cell-derived extracellular vesicles in brain disorders: mechanisms of repair and recovery

**DOI:** 10.3389/fncel.2026.1819046

**Published:** 2026-05-08

**Authors:** Masahito Nakazaki, Karen L. Lankford, Ryo Ukai, Ryosuke Hirota, Shinichi Oka, Masanori Sasaki, Jeffery D. Kocsis, Osamu Honmou

**Affiliations:** 1 Department of Neural Regenerative Medicine, School of Medicine, Institute of Regenerative Medicine, Sapporo Medical University, Sapporo, Hokkaido, Japan; 2 Department of Neurology, Yale University School of Medicine, New Haven, CT, United States; 3 Center for Neuroscience and Regeneration Research, VA Connecticut Healthcare System, West Haven, CT, United States; 4 Department of Orthopaedic Surgery, School of Medicine, Sapporo Medical University, Sapporo, Japan; 5 Division of Neuroscience, Department of Physiology, Sapporo Medical University School of Medicine, Sapporo, Hokkaido, Japan

**Keywords:** exosomes, extracellular vesicles, mesenchymal stromal/stem cells, neuroinflammation, neuronal repair, stroke, traumatic brain injury

## Abstract

Mesenchymal stem/stromal cell-derived small extracellular vesicles (MSC-sEVs) have emerged as promising cell-free therapeutics for central nervous system (CNS) disorders including stroke, traumatic brain injury (TBI), dementia, and multiple sclerosis (MS). MSC-sEVs offer advantages of low immunogenicity, ease of storage, and ability to cross the blood-brain barrier. This review provides a comprehensive analysis of the mechanisms by which MSC-sEVs have been reported to promote neural repair and recovery in preclinical models, through two convergent categories of action. First, MSC-sEVs exert direct neurorestorative effects, including activation of endogenous neural stem cells via Wnt/beta-catenin and PI3K/Akt/mTOR signaling, neuroprotection through PTEN/Akt-mediated anti-apoptotic and antioxidant pathways, preservation of mitochondrial function through mitophagy regulation, and promotion of neurite outgrowth and synaptogenesis through cytoskeletal remodeling and growth signaling. Second, MSC-sEVs modulate the injury microenvironment by shifting microglia and infiltrating macrophages toward anti-inflammatory phenotypes through NF-kB pathway modulation, converting reactive astrocytes to neuroprotective states, promoting angiogenesis and blood-brain barrier restoration, and enhancing oligodendrogenesis and remyelination. These effects are mediated largely through the transfer of microRNAs and other bioactive cargo to target cells at the injury site, although the relative contribution of individual cargo components remains to be fully established. We discuss how these actions address the pathophysiology of stroke, Alzheimer's disease, vascular dementia, TBI, and MS, highlighting disease-specific mechanisms and the current gap between preclinical evidence and clinical validation. Finally, we address challenges for clinical translation, including standardization of critical quality attributes and potency assays, route-dependent biodistribution, safety considerations, and dosing optimization. We also discuss engineering strategies for enhanced efficacy, including surface modification for CNS-targeted delivery, source cell preconditioning, cargo engineering, and scaffold-based sustained release systems. Although no clinical trials have yet evaluated MSC-sEV therapy specifically for neurological disorders, the growing body of safety data from non-neurological MSC-sEV trials and the extensive clinical experience with parent MSC therapies provide a foundation for future CNS-focused studies. MSC-sEVs hold substantial potential as a cell-free approach for neurological disorders that currently lack effective regenerative therapies, although realization of this potential will require rigorous clinical validation.

## 1 Introduction

Neurological disorders, including stroke, traumatic brain injury (TBI), dementia, and multiple sclerosis (MS), represent a major global health burden with limited therapeutic options (GBD 2019 Stroke Collaborators, 2021; GBD 2019 Dementia Forecasting Collaborators, 2022; Hajivalili et al., 2025). The central nervous system (CNS) has a limited capacity for self-repair, and current pharmacological interventions primarily target symptom management rather than tissue regeneration (Bhatti et al., 2021). This clinical reality has driven intensive research into regenerative medicine approaches, with mesenchymal stem/stromal cells (MSCs) emerging as one of the most extensively investigated candidates for neurorestorative therapy (Uccelli et al., 2011; Tan et al., 2024). Intravenous (IV) infusion of bone marrow-derived MSCs has demonstrated therapeutic efficacy in experimental models of various neurological conditions, including spinal cord injury (Osaka et al., 2010; Matsushita et al., 2011; Morita et al., 2016), stroke (Nakazaki et al., 2017; Kiyose et al., 2021), traumatic brain injury (Zhang et al., 2022), and cognitive impairment (Nakazaki et al., 2019). MSC treatment has been shown to reduce lesion volume, accelerate restoration of blood-brain barrier (BBB) and blood-spinal cord barrier (BSCB) integrity, confer neuroprotection, promote neovascularization, and modulate inflammatory responses.

Translation of these preclinical findings to clinical application has progressed substantially, particularly for acute CNS injuries. In stroke, Honmou et al. (2011) conducted a phase 2 study in which 12 patients with ischemic gray matter, white matter, and mixed lesions received IV infusion of autologous MSCs expanded in autologous human serum 36–133 days post-stroke, demonstrating safety and feasibility with no adverse events (Honmou et al., 2011). A recent systematic review and meta-analysis of randomized controlled trials confirmed the safety of IV MSC therapy for ischemic stroke and reported improvements in functional outcomes including modified Rankin Scale and Barthel Index scores, although heterogeneity across studies and small sample sizes warrant larger confirmatory trials (Hassanein et al., 2023). In spinal cord injury, Honmou et al. (2021) reported a phase 2 clinical trial involving 13 patients who received a single IV infusion of autologous MSCs cultured in autologous serum. No serious adverse events were observed, and neurological improvement based on American Spinal Injury Association (ASIA) grade was achieved in 12 of 13 patients at 6 months post-infusion. Remarkably, five of six ASIA A (complete) patients improved to ASIA B or C, and all five ASIA C patients improved to ASIA D within a few days following MSC infusion (Honmou et al., 2021). This therapeutic product (Stemirac^®^) subsequently received conditional, time-limited approval for clinical use as a human-derived somatic stem cell product in Japan. For traumatic brain injury, while clinical trials remain in earlier stages, promising preclinical results continue to support the potential of MSC-based therapies (Yamaki et al., 2024; Lu et al., 2026).

Beyond acute CNS injuries, MSC therapy is also being explored for neurodegenerative conditions, which present a fundamentally different therapeutic challenge involving chronic, progressive pathology rather than acute tissue damage. In Alzheimer's disease, the first phase 2A trial of ischemia-tolerant MSCs was granted investigational new drug approval by the US Food and Drug Administration in 2015 (Hunsberger et al., 2016). More recently, the CLEAR MIND phase 2a randomized controlled trial demonstrated that laromestrocel, an allogeneic bone marrow-derived MSC therapy, met its primary safety endpoint and showed encouraging efficacy signals in mild AD, including attenuation of brain atrophy and reduction of neuroinflammation markers (Rash et al., 2025). This program has received FDA Regenerative Medicine Advanced Therapy (RMAT) and Fast Track designations, with a pivotal phase 2/3 trial planned.

Despite the promising outcomes in both animal models of CNS injury or disease, and early clinical studies, the underlying mechanisms of MSC therapy were initially poorly understood. Notably, IV-delivered MSCs were found to not appreciably traffic to the injury site in the CNS, instead, lodging in the lungs where they survived for only 2–3 days (Quertainmont et al., 2012; Matsushita et al., 2015). This observation indicated that the therapeutic effects of MSCs were mediated predominantly through circulation of substances released by the MSCs, rather than cellular engraftment at the lesion site. Among the various factors released by MSCs, small extracellular vesicles (sEVs) have emerged as the presumptive primary mediators of MSC therapeutic action. It is important to note, however, that while the clinical data described above pertain to MSC cellular injection, the hypothesis that sEVs mediate these therapeutic effects is supported by preclinical evidence showing that MSC-sEVs replicate the effects of MSCs in many animal models of neurological injury or disease (Nakazaki et al., 2021, 2024, 2026b) and this assumption has not yet been directly validated in clinical trials of MSC-sEV therapy for neurological disorders.

Mesenchymal stromal/stem cell-derived small extracellular vesicles (MSC-sEVs) represent heterogeneous populations of membrane-bound particles with diameters of approximately 30–200 nm (Welsh et al., 2024), which are typically enriched in exosomes, but also include other subtypes of vesicles. Exosomes are a subclass of sEVs of endosomal origin that are enriched in tetraspanin proteins such as CD63, CD81, and CD9 (Welsh et al., 2024) that are produced and released by all cell types. These nano-sized vesicles carry complex cargos of proteins, lipids, and nucleic acids (including microRNAs), which are determined by the cell of origin and their environment and can mediate intercellular communication (Phinney and Pittenger, 2017).

Given their diverse bioactive cargo and ability to cross the blood-brain barrier (Alvarez-Erviti et al., 2011; Lankford et al., 2018), MSC-sEVs are now considered key mediators of the therapeutic effects observed following MSC treatment in experimental models of stroke (Xin et al., 2013a; Doeppner et al., 2015), traumatic brain injury (Zhang et al., 2015; Ni et al., 2019), neurodegenerative diseases (Reza-Zaldivar et al., 2019), and demyelinating disorders such as MS (Jafarinia et al., 2024). Accumulating evidence suggests that MSC-sEVs promote endogenous neural repair through two principal categories of action. First, MSC-sEVs act directly on neural cells: promoting neuronal survival through miR-21-mediated anti-apoptotic signaling (Han et al., 2014; Gao et al., 2020) and miR-25-mediated antioxidant protection (Zhao et al., 2019), regulating autophagy and preserving mitochondrial function via PINK1/Parkin-mediated mitophagy (Zhang et al., 2023), enhancing neurite outgrowth through miR-133b transfer (Xin et al., 2012; Li et al., 2018) and miR-17-92 cluster-mediated synaptogenesis (Xin et al., 2017), and stimulating endogenous neural stem cell activation and differentiation through miR-124-driven suppression of REST (Yang et al., 2017). Second, MSC-sEVs modulate the non-neuronal cellular microenvironment by reprogramming macrophages and microglia toward anti-inflammatory phenotypes (Lankford et al., 2018; Nakazaki et al., 2021), modulating astrocyte reactivity (Xian et al., 2019), promoting angiogenesis and BBB restoration (Nakazaki et al., 2021; Zhang et al., 2021a), and facilitating oligodendrocyte maturation and remyelination (Xin et al., 2017; Go et al., 2021), thereby creating a permissive environment conducive to neuronal restoration.

This review will describe the origin and characteristics of MSC-sEVs and examine both direct effects of MSC-sEVs on neurons—including neural stem cell activation, neuroprotection, autophagy and mitochondrial regulation, and neurite outgrowth—and indirect effects of MSC-sEVs on neurons through microenvironment modulation, encompassing immunomodulation, astrocyte phenotype conversion, angiogenesis, and remyelination. We then discuss how these actions may affect the pathophysiology of stroke, Alzheimer's disease, vascular dementia, traumatic brain injury, and multiple sclerosis, and consider challenges for clinical translation alongside emerging engineering strategies.

## 2 Origin, characteristics and cargo of MSC-sEVs

### 2.1 Mesenchymal stromal/stem cells

Mesenchymal stromal/stem cells (MSCs) are multipotent stromal cells capable of differentiating into a variety of cell types, including osteoblasts, chondrocytes, and adipocytes under appropriate conditions (Dominici et al., 2006). MSCs were initially identified in bone marrow (BM-MSCs), which remains the most extensively characterized source for both research and clinical applications (Pittenger et al., 1999). Subsequently, MSCs have been isolated from numerous other tissues, including adipose tissue (AD-MSCs), umbilical cord blood and Wharton's jelly (UC-MSCs), placenta, dental pulp, and amniotic fluid (Hass et al., 2011). Each source exhibits distinct advantages: BM-MSCs offer well-established isolation protocols and extensive clinical safety data for other conditions; AD-MSCs provide higher yields with less invasive harvesting procedures; and UC-MSCs demonstrate superior proliferative capacity, lower immunogenicity, and reduced senescence compared to adult tissue-derived MSCs (Kern et al., 2006; Jin et al., 2013). Despite differences in isolation methods, proliferation rates, and differentiation potential, MSCs from all sources share the minimal criteria established by the International Society for Cellular Therapy: plastic adherence in culture, expression of specific surface markers (CD73, CD90, CD105) with absence of hematopoietic markers (CD34, CD45, HLA-DR), and trilineage differentiation capacity (Dominici et al., 2006).

### 2.2 MSC-sEV biogenesis and characteristics

The recognition that MSCs exert their therapeutic effects primarily through secreted factors has shifted attention toward identifying the critical mediators. The MSC secretome, including cytokines, and growth factors, small extracellular vesicles (sEVs) have emerged as particularly important carriers of therapeutic cargo (Bruno et al., 2012; Phinney and Pittenger, 2017). As described above, MSC-sEVs are lipid bilayer-delimited particles in the 30–200 nm size range (Welsh et al., 2024). According to the guidelines established by the International Society for Extracellular Vesicles (ISEV) for Minimal Information for Studies of Extracellular Vesicles 2023 (MISEV2023), the term “sEVs” refers to heterogeneous vesicle populations, including exosomes and other subtypes and definitive assignment as exosomes requires demonstration of endosomal origin markers (Welsh et al., 2024). MISEV2023 recommends using operational terms based on physical characteristics (size, density), biochemical composition, or cell of origin rather than biogenesis pathway, unless the pathway has been specifically demonstrated (Welsh et al., 2024). Exosomes, which constitute a major subpopulation of sEVs, are generated through the endosomal pathway, where multivesicular bodies (MVBs) fuse with the plasma membrane releasing exosomes into the extracellular space (Colombo et al., 2014). According to MISEV2023 criteria, MSC-sEVs should be characterized by at least one protein from each of three categories: (1) transmembrane/GPI-anchored proteins (e.g., tetraspanins CD63, CD81, CD9), (2) cytosolic proteins (e.g., Alix, TSG101, syntenin), and (3) purity controls demonstrating absence of non-EV co-isolated components (Welsh et al., 2024). Western blot analysis has confirmed that MSC-sEV preparations are highly enriched in these exosomal surface markers when compared to equivalent protein amounts from the parental MSCs (Nakazaki et al., 2021, 2023). It is important to note that “exosomes” and “sEVs” are not synonymous. Exosomes are specifically defined as vesicles of endosomal origin, generated through the multivesicular body pathway, whereas the broader term “sEVs” also encompasses small ectosomes shed directly from the plasma membrane. Current isolation methods (e.g., ultracentrifugation, size-exclusion chromatography) cannot reliably separate these subtypes, and definitive assignment as exosomes requires demonstration of endosomal biogenesis markers beyond the commonly used tetraspanins (Welsh et al., 2024). As this review discusses studies with varying degrees of vesicle characterization, we refer to all as MSC-sEVs throughout for consistency with MISEV2023 recommendations. In some of the original manuscripts, these vesicles are identified as exosomes; we respect the terminology used by the original authors but note that definitive demonstration of endosomal origin has not been provided in most studies.

### 2.3 Role of sEVs in the therapeutic effects of MSCs

Mechanistically, MSCs appear to exert their therapeutic effects through released sEVs, rather than participating directly in tissue repair. Systemic administration of MSC-sEVs has been shown to promote functional recovery and neurovascular plasticity in stroke models (Xin et al., 2013a), demonstrating that sEVs alone can exert significant therapeutic effects in CNS injury. Crucially, direct comparisons in spinal cord injury models have demonstrated that fractionated MSC-sEV administration achieves functional outcomes equivalent to MSC transplantation itself, with both treatments promoting barrier function restoration and locomotor recovery (Nakazaki et al., 2021). Collectively, these observations support a model in which MSC-sEVs serve as the principal effectors of MSC-based therapy, capable of independently mediating therapeutic benefits.

**Figure 1:**
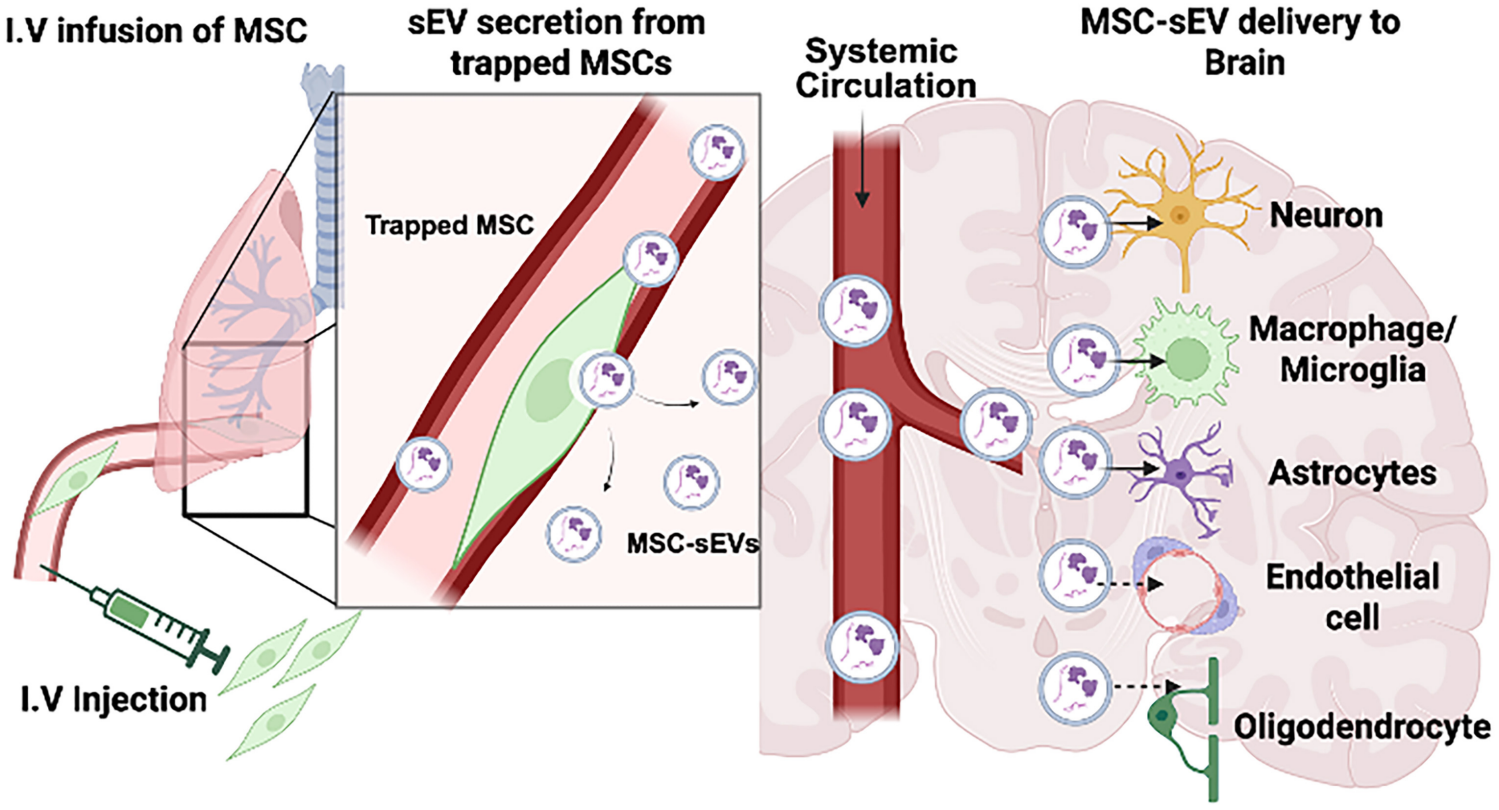
Mechanism of MSC-sEV delivery to the CNS following intravenous MSC infusion. Intravenously administered MSCs lodge transiently in the pulmonary vasculature for approximately 2–3 days, continuously releasing small extracellular vesicles (MSC-sEVs) into the systemic circulation. These MSC-sEVs traffic to sites of CNS injury and cross the compromised blood–brain barrier. *In vivo* tracking studies using fluorescently labeled sEVs have confirmed direct internalization by neurons, microglia/macrophages, and astrocytes (solid arrows). Functional evidence further implicates engagement with endothelial cells and oligodendrocytes (dashed arrows), although direct visualization of single-cell uptake *in vivo* remains to be demonstrated for these cell types. MSC, mesenchymal stem cell; sEV, small extracellular vesicle; CNS, central nervous system; BBB, blood–brain barrier.

Intravenously administered MSCs lodge transiently in the pulmonary vasculature, where they survive for approximately 2–3 days and can function as “biological factories” that continuously release sEVs into the systemic circulation (Matsushita et al., 2015; Lankford et al., 2018; Nakazaki et al., 2021). Moreover, *in vivo* tracking studies using fluorescently labeled sEVs have provided direct evidence of MSC-sEV internalization by specific cell types in the injured CNS (Figure 1). MSC-sEVs labeled with GFP, PKH26, or DiR colocalized with NeuN^+^ neurons and GFAP^+^/S100β^+^ astrocytes in stroke and traumatic brain injury models (Xin et al., 2013a; Zhang et al., 2015; Xian et al., 2019), and with Iba1^+^ microglia across broad forebrain regions (Kodali et al., 2019; Thomi et al., 2019; Losurdo et al., 2020) and M2 macrophages in contusive SCI (Lankford et al., 2018). Although direct *in vivo* uptake by oligodendrocytes and endothelial cells has not yet been visualized at the single-cell level *in vivo*, it has been observed in cell culture and functional evidence strongly implicates their involvement in MSC-sEVs therapeutic effects. MSC-sEV treatment in rodent models of stroke and spinal cord injury promotes oligodendrogenesis and remyelination (Otero-Ortega et al., 2017; Xin et al., 2021) and enhances cerebral angiogenesis with increased vWF^+^ endothelial cell density (Xin et al., 2013b). These circulating sEVs thus traffic to sites of CNS injury, cross the compromised blood-brain barrier, and deliver their bioactive cargo to neurons, astrocytes, microglia/macrophages, and likely oligodendrocytes and endothelial cells within the lesion microenvironment.

### 2.4 MSC-sEV cargo composition

The cargos of MSC-sEVs are complex, including proteins, mRNAs, microRNAs (miRNAs), circular RNAs (circRNAs), long non-coding RNAs (lncRNAs), and lipids, each with the potential to alter the phenotypic expression of recipient cells (Phinney and Pittenger, 2017). Among these, miRNAs have been proposed as key mediators of MSC-sEV therapeutic effects. Each miRNA regulates numerous mRNA targets, reshaping rates of mRNA translation and decay and profoundly altering gene expression, allowing small quantities of miRNAs to exert outsized effects on cell functions (Xin et al., 2012). Experiments showing that RNase treatment of MSC-sEVs abolished their protective effects on kidney ischemia-reperfusion injury argued that RNAs are likely a key active component (Bruno et al., 2012). As emphasized by MISEV2023, the broader compositional architecture of the sEV cargo, rather than any single component, is more likely to determine therapeutic efficacy (Welsh et al., 2024).

## 3 Direct effects of MSC-sEVs on neural cells

**Figure 2:**
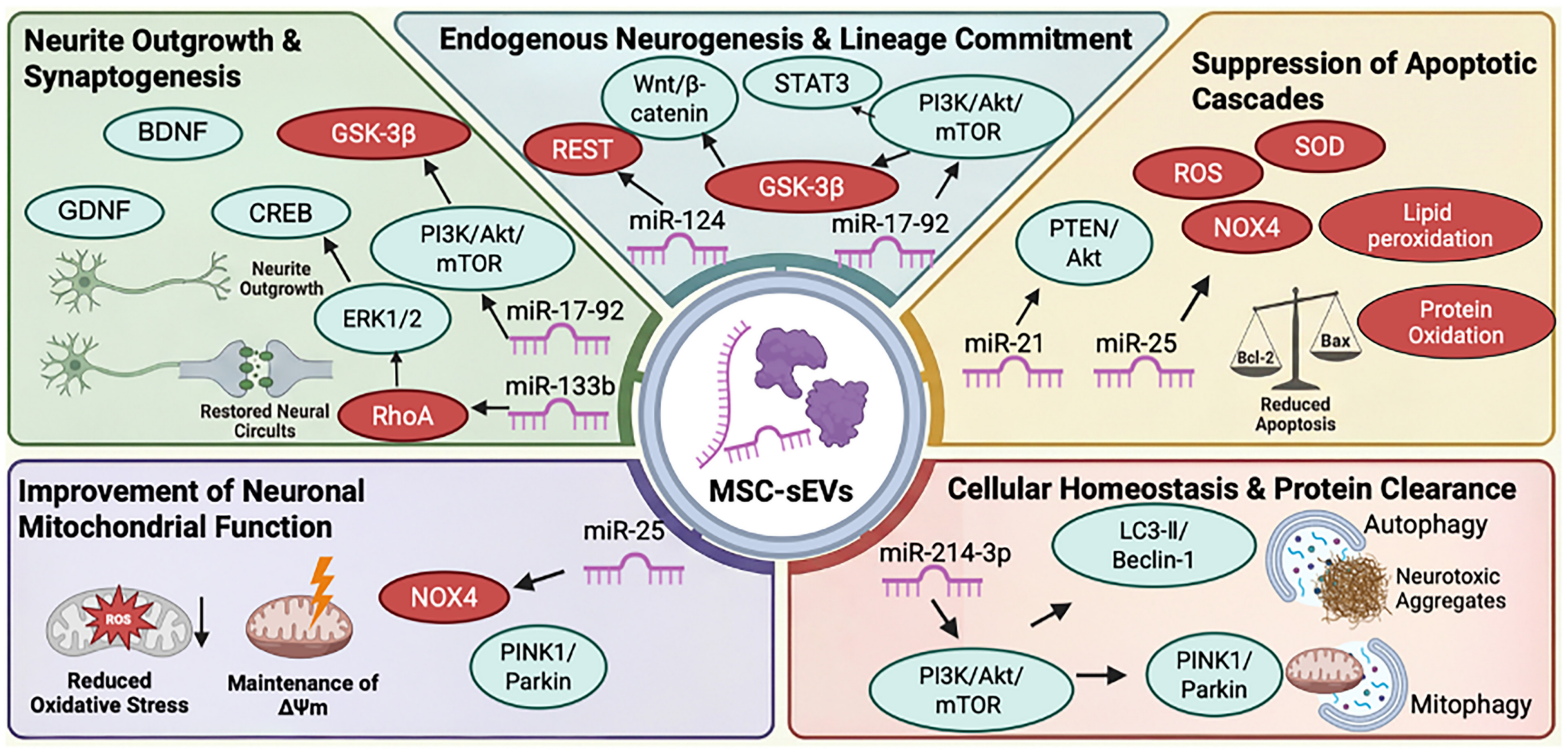
Direct neurorestorative mechanisms of MSC-sEVs on neural cells. MSC-sEVs promote neural repair through five interconnected mechanisms, each mediated by specific miRNA-target interactions and downstream signaling pathways. (1) Endogenous neurogenesis: miR-124/REST suppression and Wnt/PI3K/Akt/mTOR activation drive NSC proliferation and neuronal differentiation. (2) Anti-apoptotic neuroprotection: miR-21/PTEN/Akt and miR-25/NOX4 pathways protect neurons from apoptosis and oxidative stress. (3) Autophagy regulation: miR-214-3p/PTEN and PINK1/Parkin-mediated mitophagy maintain cellular homeostasis. (4) Mitochondrial preservation: NOX4 inhibition and PINK1/Parkin activation preserve mitochondrial membrane potential. (5) Neurite outgrowth: miR-133b/RhoA and miR-17-92/GSK-3beta promote axonal extension and dendritic remodeling. See Sections 3.1-3.5 and Table 1 for details. miR, microRNA; REST, RE1-silencing transcription factor; PI3K, phosphoinositide 3-kinase; Akt, protein kinase B; mTOR, mammalian target of rapamycin; NSC, neural stem cell; PTEN, phosphatase and tensin homolog; NOX4, NADPH oxidase 4; PINK1, PTEN-induced kinase 1; RhoA, Ras homolog family member A; GSK-3beta, glycogen synthase kinase-3beta; BDNF, brain-derived neurotrophic factor; GDNF, glial cell line-derived neurotrophic factor.

MSC-sEVs have been proposed to directly influence activation of endogenous neural stem cells, neuronal fate commitment, and the promotion of neurite outgrowth and synaptogenesis (See Figure 2).

### 3.1 Activation and differentiation of endogenous neural stem cells

Neural stem cells (NSCs) reside in specialized niches within the adult brain, primarily the subventricular zone (SVZ) and the subgranular zone (SGZ) of the hippocampal dentate gyrus, where they can be activated to generate new neurons throughout life (Ming and Song, 2011). Under pathological conditions such as stroke and traumatic brain injury, endogenous NSCs can be mobilized to contribute to neural repair, although this intrinsic regenerative capacity is often insufficient for functional recovery (Arvidsson et al., 2002; Yamashita et al., 2006). MSC-sEVs have been shown to enhance endogenous neurogenesis by promoting both NSC proliferation and differentiation into mature neurons (Zhang et al., 2015; Yang et al., 2017).

MSC-sEVs activate multiple signaling pathways that regulate NSC proliferation. The Wnt/β-catenin pathway, a master regulator of NSC self-renewal, can be modulated by MSC-sEV-derived miRNAs (Xin et al., 2017). Treatment with MSC-sEVs has been shown to increase the expression of doublecortin (DCX), a marker of immature neurons, and enhance neuroblast migration toward injury sites in stroke models (Xin et al., 2013a). The miR-17-92 cluster, enriched in MSC-sEVs, has been reported to promote oligodendrogenesis, neurogenesis, and axonal outgrowth by targeting PTEN and activating the PI3K/Akt/mTOR signaling pathway in rodent stroke models (Xin et al., 2017). Additionally, MSC-sEVs have been reported to stimulate NSC proliferation through the transfer of growth factors and cytokines that activate the STAT3 and MAPK/ERK pathways (Qiu et al., 2018).

Beyond proliferation, MSC-sEVs also promote neuronal fate commitment, directing neural progenitor cells to differentiate into mature neurons. This process requires the suppression of non-neuronal gene programs and activation of neuron-specific transcription. miR-124, one of the most abundant miRNAs in the brain, has been proposed to play a central role in neuronal differentiation by suppressing the RE1-silencing transcription factor (REST), a master repressor of neuronal gene expression in non-neuronal cells (Conaco et al., 2006). REST suppression by miR-124 de-represses hundreds of neuron-specific genes, including ion channels, neurotransmitter receptors, and synaptic proteins, thereby driving progenitor cells toward a neuronal identity. MSC-sEVs enriched with miR-124 have been shown to enhance neuronal differentiation and functional recovery in stroke models (Yang et al., 2017). Although this mechanism is well-characterized *in vitro*, its contribution to the therapeutic effects of MSC-sEVs *in vivo* remains to be established.

### 3.2 Anti-apoptotic effects

MSC-sEVs also exert critical neuroprotective effects on existing mature neurons. Following CNS injury, neurons are exposed to multiple death-inducing stimuli, including excitotoxicity, oxidative stress, inflammation, and mitochondrial dysfunction (Alizadeh et al., 2019; Liu et al., 2025). MSC-sEVs have been shown to protect neurons against apoptosis through several distinct mechanisms.

miR-21, highly expressed in MSC-sEVs, has been shown to exert anti-apoptotic effects in neurons in several preclinical studies (Han et al., 2014; Gao et al., 2020). Han et al. (2014) demonstrated that miR-21 alleviates apoptosis of cortical neurons through activation of the PTEN-Akt signaling pathway after traumatic brain injury (Han et al., 2014). More recently, Gao et al. (2020) showed that MSC-derived extracellular vesicles transfer miR-21-5p directly to neurons, alleviating early brain injury and improving cognitive function via the PTEN/Akt pathway after subarachnoid hemorrhage (Gao et al., 2020). Additionally, MSC-sEV-derived miR-21 contributes to neuroprotection indirectly by modulating neuroinflammation; Li et al. (2025) demonstrated that miR-21-5p/PDCD4-mediated shifting of macrophage M1/M2 polarization promotes functional recovery in spinal cord injury (Li et al., 2025). Additionally, MSC-sEVs modulate the balance of Bcl-2 family proteins, increasing the anti-apoptotic Bcl-2 while decreasing the pro-apoptotic Bax, thereby stabilizing mitochondrial membrane integrity and preventing cytochrome c release (Xin et al., 2013a).

MSC-sEVs also protect neurons from oxidative stress-induced apoptosis. Exosomes derived from MSCs overexpressing miR-25 protect spinal cord neurons against ischemia-reperfusion injury by inhibiting NADPH oxidase 4 (NOX4), a major source of reactive oxygen species (ROS), thereby reducing malondialdehyde content and increasing superoxide dismutase (SOD) activity (Zhao et al., 2019). This antioxidant capacity helps prevent lipid peroxidation and protein oxidation that trigger apoptotic pathways following ischemic injury.

### 3.3 Neuronal autophagy and mitophagy regulation

Autophagy is an evolutionarily conserved cellular process essential for neuronal homeostasis, degrading damaged organelles, misfolded proteins, and aggregated proteins that accumulate following CNS injury (Hara et al., 2006; Komatsu et al., 2006). Unlike proliferating cells, post-mitotic neurons are particularly dependent on efficient autophagy to maintain cellular health throughout their lifespan (Hara et al., 2006). Following CNS injury, dysregulated autophagy in neurons contributes to cell death and dysfunction, making it a critical therapeutic target (Komatsu et al., 2006). The PI3K/Akt/mTOR signaling axis, a central regulator of autophagy, is modulated by MSC-sEV-derived miRNAs in neurons. Following ischemic injury, PTEN (phosphatase and tensin homolog) expression is progressively upregulated in the penumbra zone, leading to excessive suppression of the pro-survival Akt pathway (Wu et al., 2024). Wu et al. (2024) demonstrated that hypoxia-preconditioned MSC-derived exosomes deliver miR-214-3p, which directly targets and downregulates PTEN expression, thereby restoring appropriate Akt activation and improving neurological function after stroke (Wu et al., 2024). This mechanism exemplifies how MSC-sEVs achieve pathway balance: under pathological conditions where PTEN overexpression impairs neuronal survival signaling, miR-214-3p-mediated PTEN suppression re-establishes the equilibrium necessary for neuroprotection. MSC-sEVs also enhance the clearance of neurotoxic protein aggregates through autophagy enhancement (Wu et al., 2024). In models of CNS injury, damaged neurons accumulate misfolded proteins and aggregates that contribute to secondary injury. MSC-sEV treatment has been shown to increase the expression of autophagy markers LC3-II and Beclin-1 in injured neurons while decreasing p62/SQSTM1, indicating enhanced autophagic flux and efficient clearance of damaged cellular components (Liu et al., 2025). This autophagic clearance function is particularly relevant for preventing the chronic neurodegeneration that can follow acute CNS injury.

### 3.4 Improvement of neuronal mitochondrial function

Mitochondrial dysfunction is a hallmark of secondary injury following CNS trauma, contributing to energy failure, oxidative stress, and neuronal death (Harrison et al., 2016; Liu et al., 2025). Neurons are particularly vulnerable to mitochondrial impairment due to their high energy demands and limited glycolytic capacity. Following injury, damaged mitochondria produce excessive reactive oxygen species (ROS), lose membrane potential, and release pro-apoptotic factors such as cytochrome c (Harrison et al., 2016; Liu et al., 2025). MSC-sEVs have emerged as potent modulators of neuronal mitochondrial function, protecting against these pathological processes through multiple mechanisms. MSC-sEVs protect neuronal mitochondria by reducing oxidative stress. As discussed in Section 3.2, miR-25-enriched MSC-sEVs inhibit NADPH oxidase 4 (NOX4), a major source of ROS, thereby reducing oxidative damage to mitochondrial membranes and preserving mitochondrial integrity (Zhao et al., 2019). Additionally, MSC-sEVs have been shown to restore the balance between pro-oxidant and antioxidant systems in injured neurons, increasing superoxide dismutase (SOD) activity and reducing malondialdehyde levels, a marker of lipid peroxidation (Ni et al., 2019). MSC-sEVs also maintain mitochondrial membrane potential, which is essential for ATP production through oxidative phosphorylation (Zhang et al., 2025). Loss of mitochondrial membrane potential is an early event in neuronal apoptosis, triggering the release of cytochrome c and activation of caspases. Zhang et al. (2023) demonstrated that hUC-MSC-derived exosomes preserve mitochondrial membrane potential in injured neurons by activating the PINK1/Parkin pathway, which facilitates the selective removal of depolarized mitochondria through mitophagy before they can trigger apoptotic cascades (Zhang et al., 2023). This mechanism ensures that the remaining mitochondrial population maintains functional integrity.

The neuroprotective effects of MSC-sEVs on mitochondrial function have been validated in large animal models. Williams et al. (2020) demonstrated in a swine model of traumatic brain injury and hemorrhagic shock that early single-dose treatment with MSC-derived exosomes significantly attenuated brain swelling and lesion size while improving blood-brain barrier integrity (Williams et al., 2020). These protective effects are likely mediated, in part, through preservation of neuronal mitochondrial function during the critical early period following injury when secondary injury mechanisms are most active.

### 3.5 Neurite outgrowth and synaptogenesis

The promotion of neurite outgrowth and synaptogenesis is essential for restoring neural connectivity and function after CNS injury (Xin et al., 2012, 2013a; Zhang et al., 2015; Li et al., 2018). MSC-sEVs have been demonstrated to enhance axonal regeneration and synaptic plasticity through multiple mechanisms. miR-133b has been identified as a candidate mediator of MSC-sEV-induced neurite outgrowth. Xin et al. (2012) first demonstrated that MSC-derived exosomes transfer miR-133b to neural cells, promoting neurite outgrowth *in vitro* (Xin et al., 2012). Subsequent studies showed that intravenous administration of miR-133b-enriched MSC-sEVs significantly improved hindlimb locomotor recovery after spinal cord injury by reducing RhoA expression, a negative regulator of axonal growth, and activating ERK1/2, STAT3, and CREB signaling pathways (Li et al., 2018). RhoA inhibition allows for cytoskeletal reorganization and growth cone advancement, facilitating axonal extension toward target neurons (Li et al., 2018). The miR-17-92 cluster delivered by MSC-sEVs also contributes to neurite remodeling and neural plasticity. Xin et al. (2017) demonstrated that treatment with miR-17-92 cluster-enriched exosomes significantly enhanced dendritic remodeling and increased dendritic spine density in the peri-infarct region after stroke (Xin et al., 2017). This was associated with increased phosphorylation of Akt, mTOR, and GSK-3β, suggesting activation of pro-growth signaling pathways. Furthermore, MSC-sEVs have been shown to increase the expression of synaptic proteins such as synaptophysin and PSD-95, indicating enhanced synaptogenesis (Doeppner et al., 2015).

Beyond miRNA transfer, MSC-sEVs contain proteins that directly support neurite outgrowth, including neurotrophic factors such as BDNF and NGF, as well as extracellular matrix proteins that provide a permissive substrate for axonal growth (Harrell et al., 2019). The combined delivery of multiple bioactive molecules by MSC-sEVs creates a comprehensive pro-regenerative environment that supports all stages of neural circuit repair.

It should be noted that the mechanistic links described above are derived predominantly from individual preclinical studies, often using engineered miRNA overexpression systems in rodent models. The relative contribution of any single miRNA to the overall therapeutic effects of unmodified MSC-sEVs *in vivo* remains to be determined, and as emphasized by MISEV2023, the broader compositional architecture of the sEV cargo, rather than any individual component, is more likely to determine therapeutic efficacy (Welsh et al., 2024).

## 4 Modulation of neuronal microenvironment by MSC-sEVs

**Figure 3:**
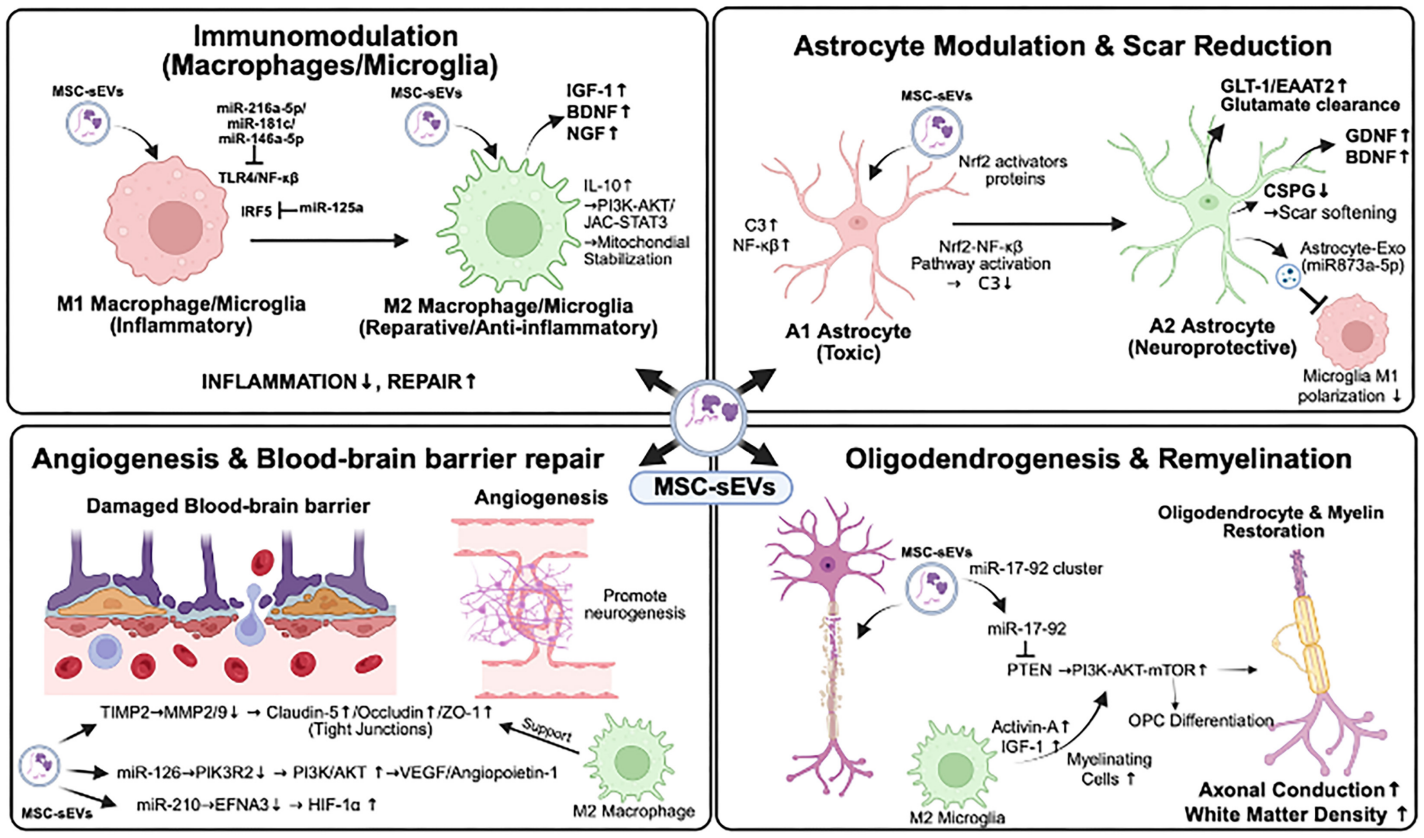
Mechanisms of MSC-sEV-mediated modulation of neuronal microenvironment. MSC-sEVs remodel the injury microenvironment through four mechanisms, each involving specific miRNA-mediated signaling cascades and cellular interactions. (1) Immunomodulation: multiple miRNAs (miR-216a-5p, miR-181c, miR-146a-5p, miR-125a) converge on NF-kB/IRF5 suppression to shift macrophages/microglia from M1 toward M2 phenotypes. (2) Astrocyte modulation: Nrf2-NF-kB pathway activation converts neurotoxic A1 astrocytes to neuroprotective A2 states. (3) Angiogenesis and BBB repair: miR-126 and miR-210 promote neovascularization via PI3K/Akt and HIF-1alpha signaling, while TIMP2-mediated MMP inhibition preserves tight junction proteins. (4) Oligodendrogenesis: miR-17-92/PTEN/PI3K/Akt/mTOR signaling promotes OPC differentiation, supported by M2 microglia-derived trophic factors. See Sections 4.1-4.4 and Table 1 for details. miR, microRNA; NF-kB, nuclear factor kappa-light-chain-enhancer of activated B cells; IRF5, interferon regulatory factor 5; Nrf2, nuclear factor erythroid 2-related factor 2; PI3K, phosphoinositide 3-kinase; Akt, protein kinase B; HIF-1alpha, hypoxia-inducible factor-1alpha; TIMP2, tissue inhibitor of metalloproteinase 2; MMP, matrix metalloproteinase; PTEN, phosphatase and tensin homolog; mTOR, mammalian target of rapamycin; OPC, oligodendrocyte precursor cell; BBB, blood-brain barrier.

MSC-sEVs modulate the neuronal microenvironment through at least four distinct mechanisms, immunomodulation via macrophage/microglia polarization, promotion of angiogenesis and neurovascular coupling, and enhancement of oligodendrogenesis and remyelination (See Figure 3).

### 4.1 Immunomodulation and microglia/macrophage polarization

Following CNS injury, resident microglia and infiltrating macrophages play dual roles in tissue damage and repair (Kigerl et al., 2009; Kroner et al., 2014; Nakazaki et al., 2026b). The classically activated M1 phenotype produces pro-inflammatory cytokines (TNF-α, IL-1β, IL-6) and reactive oxygen species that exacerbate secondary injury, while the alternatively activated M2 phenotype secretes anti-inflammatory mediators (IL-10, TGF-β) and neurotrophic factors that promote tissue repair (Kigerl et al., 2009; Mantovani et al., 2013). Importantly, the M1/M2 paradigm represents a simplified conceptual framework; *in vivo*, macrophages and microglia exist along a continuous activation spectrum rather than discrete binary states, dynamically shifting phenotypes in response to local microenvironmental cues such as cytokines, damage-associated molecular patterns (DAMPs), and cell–cell interactions (Gensel and Zhang, 2015; Hu et al., 2015). This phenotypic plasticity underscores the therapeutic opportunity to redirect rather than simply suppress myeloid cell responses after CNS injury. MSC-sEVs have been shown to shift the balance from pro-inflammatory toward anti-inflammatory phenotypes, creating a microenvironment conducive to neural recovery (Liu et al., 2020; Nakazaki et al., 2021, 2023).

Several miRNAs carried by MSC-sEVs promote M1-to-M2 polarization through distinct molecular targets that converge on the TLR4/NF-κB signaling pathway (Nakazaki et al., 2026b). Exosomal miR-216a-5p derived from hypoxia-preconditioned BM-MSCs shifts microglia from M1 to M2 via TLR4/NF-κB suppression (Liu et al., 2020). Similarly, miR-181c from hUC-MSC-sEVs down-regulates TLR4 and depresses p65 activation (Zhang et al., 2021b) miR-146a-5p from hypoxia-preconditioned BM-MSC-sEVs targets TRAF6, suppressing downstream NF-κB signaling and promoting M1 → M2 macrophage polarization (Liang et al., 2024), while also inhibiting NLRP3 inflammasome-mediated pyroptosis (Hua et al., 2022). Beyond the TLR4/NF-κB axis, miR-125a down-regulates IRF5, a master transcription factor driving M1 polarization, thereby promoting M2 polarization and neuroprotection (Chang et al., 2021).

The functional consequences of M2 polarization extend beyond cytokine modulation. M2 macrophages/microglia exhibit enhanced phagocytic capacity for debris clearance and secrete neurotrophic factors that directly support neuronal survival and regeneration. Specifically, M2 macrophages deliver IGF-1 as a key neurotrophic factor (Forster et al., 2019), while both M2 macrophages and microglia produce BDNF and NGF that activate neuronal survival pathways (Kigerl et al., 2009). Additionally, M2 macrophages/microglia produce IL-10 and TGF-β; IL-10 engages PI3K-AKT and JAK-STAT3 signaling to stabilize mitochondria and suppress apoptosis (Zhou et al., 2009), while TGF-β promotes blood-brain/spinal cord barrier repair through upregulation of tight junction proteins such as ZO-1 and occludin (Nakazaki et al., 2021). Our previous work demonstrated that MSC-sEVs are preferentially internalized by CD206+ M2 macrophages at the injury site, with minimal uptake by iNOS+ M1 macrophages, suggesting that MSC-sEVs amplify the reparative capacity of cells already primed for a healing response (Lankford et al., 2018; Nakazaki et al., 2026b).

### 4.2 Astrocyte modulation and indirect neuroprotection

Astrocytes are the most abundant glial cells in the CNS and play critical roles in maintaining neuronal homeostasis. Following CNS injury, astrocytes undergo reactive astrogliosis, characterized by hypertrophy, proliferation, and GFAP upregulation (Sofroniew and Vinters, 2010; Liddelow and Barres, 2017). While initial studies proposed a binary classification of neurotoxic A1 and neuroprotective A2 astrocytes (Zamanian et al., 2012; Liddelow et al., 2017), reactive astrocytes exist along a continuous spectrum of activation states (Escartin et al., 2021). MSC-sEVs can modulate this spectrum, shifting astrocyte phenotypes from neurotoxic toward neuroprotective states.

MSC-sEVs ameliorate inflammation-induced astrocyte alterations through the Nrf2-NF-κB signaling pathway, reducing A1 marker expression (C3) while preserving normal astrocyte functions (Xian et al., 2019; Lai et al., 2022). The neuroprotective effects are mediated through multiple mechanisms: (1) maintaining glutamate transporter expression (GLT-1/EAAT2) to prevent excitotoxicity (Zhuang et al., 2022); (2) increasing neurotrophic factor production including BDNF and GDNF (Zhang et al., 2015); and (3) attenuating glial scar formation by reducing CSPG deposition. Additionally, astrocyte-microglia crosstalk contributes to MSC-sEV effects; astrocyte-derived exosomes enriched with miR-873a-5p inhibit microglial M1 polarization via NF-κB suppression (Long et al., 2020), suggesting that MSC-sEVs may prime astrocytes to produce their own therapeutic exosomes.

### 4.3 Angiogenesis, vascular protection, and neurovascular coupling

The restoration and protection of vascular supply is essential for neural repair, as neurons depend on a continuous supply of oxygen and nutrients. Following CNS injury, disruption of the blood-brain barrier (BBB) or blood-spinal cord barrier (BSCB) leads to edema, infiltration of peripheral immune cells, and chronic neuroinflammation that exacerbate neuronal death (Nakazaki et al., 2021). MSC-sEVs promote both angiogenesis and vascular barrier protection through the delivery of pro-angiogenic miRNAs and barrier-stabilizing factors. MSC-sEVs exert potent protective effects on the BBB and BSCB through multiple mechanisms. MSC-sEVs suppress matrix metalloproteinases (MMP-2/9) via the TIMP2 pathway, thereby preserving tight junction proteins such as claudin-5, occludin, and ZO-1 (Xin et al., 2021). Our previous work demonstrated that fractionated dosing of MSC-sEVs over three consecutive days, mimicking the gradual release from lung-entrapped MSCs, accelerated BSCB recovery in subacute SCI; both MSC and fractionated MSC-sEV treatment upregulated tight junction proteins (ZO-1, occludin) and the adherens junction protein N-cadherin, correlating with reduced BSCB permeability and improved functional outcomes (Nakazaki et al., 2021). These barrier-protective effects are mediated in part through TGF-β signaling, with MSC-sEV-treated M2 macrophages producing TGF-β that upregulates TGF-β receptors on the microvasculature (Nakazaki et al., 2021, 2024).

In addition to vascular protection, MSC-sEVs promote angiogenesis through the delivery of pro-angiogenic miRNAs. miR-126, an endothelial-specific miRNA associated with vascular homeostasis, has been proposed as a mediator of MSC-sEV-induced angiogenesis based on in vitro and rodent studies. Exosomes derived from miR-126-overexpressing BM-MSCs promote the proliferation, migration, and tube formation of human umbilical vein endothelial cells (HUVECs) by targeting PIK3R2 and activating the PI3K/Akt signaling pathway (Zhang et al., 2021b). This leads to upregulation of angiogenesis-related factors including VEGF and angiopoietin-1. *In vivo*, miR-126-enriched MSC-sEVs significantly increase capillary density at injury sites and accelerate wound healing. MSC-sEVs contain other pro-angiogenic factors. miR-210, known as the “master hypoxamir,” promotes angiogenesis under hypoxic conditions by targeting EFNA3 and stabilizing HIF-1α signaling (Moon et al., 2019). MSC-sEVs also deliver VEGF protein directly to endothelial cells, stimulating endothelial proliferation and migration (Doeppner et al., 2015). The combined delivery of miRNAs and proteins by MSC-sEVs creates a synergistic pro-angiogenic effect that supports the revascularization necessary for neural tissue repair.

Importantly, angiogenesis is tightly coupled with neurogenesis in the neurovascular niche (Cunningham et al., 2018). Zhang et al. (2015) demonstrated that MSC-derived exosomes promote both endogenous angiogenesis and neurogenesis after traumatic brain injury, significantly increasing the number of newborn endothelial cells and immature neurons in the dentate gyrus (Zhang et al., 2015). New blood vessels provide not only oxygen and nutrients but also secrete neurotrophic factors and guidance cues that support NSC proliferation and neuronal survival (Teng et al., 2008). The dual action of MSC-sEVs—protecting existing vasculature while promoting new vessel formation—thus contributes comprehensively to neural repair by maintaining barrier integrity and establishing a vascular scaffold that supports neural repair processes.

### 4.4 Oligodendrogenesis and remyelination

Demyelination is a common consequence of CNS injury, leading to impaired axonal conduction and neurological dysfunction. Oligodendrocytes, the myelinating cells of the CNS, are particularly vulnerable to ischemia and inflammation. MSC-sEVs have been shown to promote oligodendrocyte precursor cell (OPC) differentiation and remyelination, thereby restoring neural conduction and function. The miR-17-92 cluster, previously discussed in the context of neurogenesis (Section 3.1), also promotes oligodendrogenesis. Xin et al. (2017) demonstrated that miR-17-92 cluster-enriched exosomes enhance oligodendrocyte differentiation and increase myelin basic protein (MBP) expression in the peri-infarct region after stroke (Xin et al., 2017). This has been attributed to PTEN suppression and activation of the PI3K/Akt/mTOR pathway, which is essential for oligodendrocyte differentiation and myelin synthesis (Xin et al., 2017). Go et al. (2021) provided compelling evidence for MSC-sEV-mediated myelin maintenance in a non-human primate model of cortical injury (Go et al., 2021). Treatment with MSC-sEVs reduced the density of damaged oligodendrocytes in sublesional white matter, upregulated myelin-related genes, and increased the number of actively myelinating oligodendrocytes. These changes correlated with improved motor recovery, suggesting that enhanced myelin maintenance facilitates functional recovery after injury, particularly in the aged brain where white matter damage is prevalent.

MSC-sEVs also protect oligodendrocyte lineage cells indirectly through immunomodulation. Oligodendrocyte precursor cells (OPCs) are particularly vulnerable to inflammatory injury; TNF-α, a major product of M1 microglia/macrophages and astrocytes, is highly toxic to OPCs and inhibits their survival and differentiation into mature myelinating oligodendrocytes (Pang et al., 2005; Su et al., 2011). By shifting microglia/macrophages toward the M2 phenotype, MSC-sEVs significantly reduce TNF-α and IL-6 levels at the injury site, as demonstrated in spinal cord injury models where MSC-sEV treatment decreased pro-inflammatory cytokine production while increasing anti-inflammatory markers such as TGF-β and Arg1 (Nakazaki et al., 2023). This reduction of TNF-α in the lesion microenvironment directly alleviates the cytotoxic burden on OPCs, while M2 microglia actively promote oligodendrocyte differentiation through the secretion of trophic factors such as activin-A and IGF-1 (Miron et al., 2013). Thus, the immunomodulatory effects of MSC-sEVs create a permissive environment for OPC survival, differentiation, and remyelination.

While the microenvironment modulating effects described above are supported by multiple preclinical studies, most evidence derives from rodent injury models, and individual pathway analyses may not capture the complexity of MSC-sEV actions *in vivo*. Cross-model validation, including large animal and human-derived systems, will be important for establishing the translational relevance of these mechanisms.

## 5 CNS therapeutic applications and mechanisms of action of MSC-sEVs

MSC-sEVs impact the pathophysiologies of CNS conditions such as stroke, dementia (including Alzheimer's disease and vascular dementia), traumatic brain injury, and multiple sclerosis at multiple levels.

### 5.1 Stroke

Stroke remains a leading cause of death and disability worldwide, with ischemic stroke accounting for approximately 85% of all cases (Virani et al., 2020). The pathophysiology of ischemic stroke involves acute neuronal death due to energy failure, followed by secondary injury cascades including excitotoxicity, oxidative stress, neuroinflammation, and blood-brain barrier disruption that expand the lesion over days to weeks (Otero-Ortega et al., 2019).

MSC-sEVs can influence multiple aspects of stroke pathophysiology. In the acute phase, MSC-sEVs can have neuroprotective effects through anti-apoptotic mechanisms, including miR-21-mediated suppression of PDCD4 and miR-25-mediated inhibition of NOX4, thereby protecting neurons from ischemia-reperfusion injury (Zhao et al., 2019; Li et al., 2025). The preservation of mitochondrial function through PINK1/Parkin-mediated mitophagy is particularly relevant in stroke, where mitochondrial dysfunction drives neuronal death during the penumbral expansion phase (Zhang et al., 2023). During the subacute and chronic phases, the effects of MSC-sEVs are more complex. Hao et al. (2022) has shown that MSC-sEVs treatments shift microglia/macrophages from pro-inflammatory toward anti-inflammatory phenotypes, reducing secondary injury and creating a permissive environment for repair (Hao et al., 2022), while Xin et al. (2013a) demonstrated that MSC-sEVs enhance neurogenesis and angiogenesis while reducing inflammation. The promotion of endogenous neurogenesis through Wnt/β-catenin and PI3K/Akt/mTOR signaling increases the generation of new neurons from the subventricular zone, with neuroblasts migrating toward the peri-infarct region (Xin et al., 2013a, 2017). Both angiogenesis and oligodendrocyte differentiation and remyelination are critical for stroke recovery and potential targets of MSC-sEVs. MSC-sEV-derived miR-126 and miR-210 promote endothelial proliferation and tube formation, significantly increasing vascular density in the peri-infarct region (Moon et al., 2019). The miR-17-92 cluster delivered by MSC-sEVs enhances oligodendrocyte differentiation and increases myelin basic protein expression (Xin et al., 2013a).

Multiple preclinical studies have demonstrated significant functional improvements following MSC-sEV treatment in stroke models. Xin et al. (2013a) first reported that systemic administration of MSC-sEVs promotes functional recovery and neurovascular plasticity in a rat MCAO model (Xin et al., 2013a). Doeppner et al. (2015) subsequently demonstrated that MSC-sEVs improve post-stroke neuroregeneration and prevent postischemic immunosuppression, with effects comparable to MSC transplantation itself (Doeppner et al., 2015). Xia et al. (2020) showed that sEVs from iPSC-derived MSCs reduce tissue loss and promote long-term neurological recovery in a chronic stage stroke model (Xia et al., 2020). Additionally, Xin et al. (2013b) demonstrated that miR-133b-enriched MSC-sEVs promote neural plasticity and functional recovery after stroke via transfer to neurons and astrocytes (Xin et al., 2013b).

Taken together, these studies demonstrate that MSC-sEVs exert multifaceted therapeutic effects in stroke, potentially providing neuroprotection in the acute phase of injury through anti-apoptotic mechanisms, as well as promoting sustained recovery through immunomodulation, neurogenesis, angiogenesis, and white matter repair in later phases. The convergence of these mechanisms on both gray and white matter pathology positions MSC-sEVs as a promising therapeutic approach for ischemic stroke at multiple stages after injury. However, it should be noted that functional outcomes reported in rodent stroke models may not directly predict clinical efficacy, and disease-specific optimization of treatment parameters, including dose, timing, and route, has yet to be performed in human subjects.

### 5.2 Dementia: Alzheimer's disease and vascular dementia

Dementia represents a growing global health crisis, with Alzheimer's disease (AD) accounting for 60–70% of cases and vascular dementia (VaD) comprising the second most common form (Hernandez and Garcia, 2021; Liu et al., 2024). While AD and VaD have distinct primary etiologies—amyloid-β accumulation and neurofibrillary tangles in AD vs. cerebrovascular pathology in VaD—they share several common pathophysiological features including neuroinflammation, synaptic dysfunction, and neuronal loss that can be targeted by MSC-sEVs.

In Alzheimer's disease, MSC-sEVs address multiple pathological processes. Neuroinflammation plays a central role in AD progression, with activated microglia and astrocytes surrounding amyloid plaques and releasing pro-inflammatory cytokines that exacerbate neurodegeneration (Ding et al., 2018). MSC-sEV-mediated immunomodulation shifts microglia toward anti-inflammatory phenotypes, reducing TNF-α, IL-1β, and IL-6 while increasing IL-10 and TGF-β (Cui et al., 2019; Ding et al., 2018). Importantly, Cui et al. (2019) demonstrated that RVG-modified MSC-sEVs targeted to the brain significantly reduced amyloid plaque deposition and improved cognitive function in APP/PS1 transgenic mice, with enhanced effects compared to unmodified exosomes (Cui et al., 2019).

Astrocyte modulation is particularly relevant in AD, where reactive astrogliosis contributes to synaptic dysfunction and neuronal death (Liddelow et al., 2017). MSC-sEVs promote the transition from neurotoxic A1 to neuroprotective A2 astrocytes, maintaining glutamate homeostasis and preventing excitotoxicity (Xian et al., 2019). The neuronal autophagy-promoting effects of MSC-sEVs may also facilitate clearance of misfolded proteins including amyloid-β and phosphorylated tau (Shin et al., 2014), although further research is needed to confirm this mechanism in AD models.

The promotion of neurogenesis and synaptogenesis by MSC-sEVs is particularly important in AD, where synaptic loss correlates most closely with cognitive decline (Selkoe, 2002). Reza-Zaldivar et al. (2019) demonstrated that MSC-derived exosomes stimulated neurogenesis in the subventricular zone and alleviated amyloid-β-induced cognitive impairment, with effects comparable to MSC transplantation (Reza-Zaldivar et al., 2019). MSC-sEVs also contain neprilysin, a major amyloid-β-degrading enzyme, potentially directly reducing amyloid burden (Katsuda et al., 2013).

Vascular dementia shares many therapeutic targets with stroke, as both involve cerebrovascular pathology (Iadecola, 2013). MSC-sEV-mediated BBB protection prevents chronic vascular leakage that contributes to white matter damage and cognitive decline (Liu et al., 2024). The angiogenic effects of MSC-sEVs restore cerebral perfusion, while oligodendrogenesis promotion repairs white matter lesions that are characteristic of VaD (Liu et al., 2024). Given the frequent coexistence of AD and vascular pathology (mixed dementia), MSC-sEVs may offer particular benefit through their ability to simultaneously address both neurodegenerative and vascular mechanisms (Iadecola, 2013).

It is important to note that MSC-sEV-based approaches for dementia remain at an early preclinical stage, and the chronic, progressive nature of both AD and VaD presents challenges distinct from acute CNS injuries where MSC-sEVs treatments have been more extensively studied.

### 5.3 Traumatic brain injury

Traumatic brain injury (TBI) initiates complex secondary injury cascades that expand tissue damage over hours to weeks following the primary mechanical insult (Shahror et al., 2020). These secondary processes include excitotoxicity, oxidative stress, mitochondrial dysfunction, neuroinflammation, and BBB disruption—all of which represent targets for MSC-sEV therapy (Zhang et al., 2015; Blaya et al., 2025).

In the acute phase of TBI, MSC-sEVs provide neuroprotection through anti-apoptotic mechanisms. Williams et al. (2020) demonstrated in a large animal model of TBI and hemorrhagic shock that early single-dose MSC-sEV treatment significantly reduced brain swelling and lesion size while improving BBB integrity (Williams et al., 2020). The mitochondrial protective effects of MSC-sEVs are particularly relevant in TBI, where mitochondrial dysfunction drives secondary neuronal death through ROS generation and energy failure (Hering and Shetty, 2023). PINK1/Parkin-mediated mitophagy activated by MSC-sEVs clears damaged mitochondria, preventing the accumulation of toxic metabolites (Zhang et al., 2023).

Neuroinflammation following TBI involves both resident microglia and infiltrating macrophages, which can either exacerbate tissue damage through pro-inflammatory mediator release or promote repair through debris clearance and neurotrophic factor secretion (Chen et al., 2020). MSC-sEV-mediated immunomodulation shifts these cells toward anti-inflammatory phenotypes. Ni et al. (2019) demonstrated that BM-MSC-derived exosomes modulate microglia/macrophage polarization by downregulating iNOS and upregulating CD206 and Arg1, thereby reducing early neuroinflammation after TBI (Ni et al., 2019).

Astrocyte responses to TBI also influence outcome. While reactive astrogliosis is initially protective, excessive or prolonged astrogliosis contributes to glial scar formation that impedes axonal regeneration (Liu et al., 2020). MSC-sEVs modulate astrocyte phenotype, reducing neurotoxic A1 astrocytes and promoting neuroprotective A2 astrocytes that support neuronal survival and maintain BBB integrity (Xian et al., 2019; Liu et al., 2020).

In addition to acute neuroprotection, angiogenesis and neurogenesis are essential for long-term functional recovery after TBI. Zhang et al. (2015) demonstrated that MSC-derived exosomes improved cognitive and sensorimotor function in rats after TBI by promoting endogenous angiogenesis and neurogenesis while reducing neuroinflammation (Zhang et al., 2015). The neurite outgrowth-promoting effects of MSC-sEVs, mediated through miR-133b and the miR-17-92 cluster, enhance axonal regeneration and synaptic connectivity (Xin et al., 2012, 2017). Furthermore, oligodendrogenesis promotion supports white matter repair, which is particularly important given that diffuse axonal injury and white matter pathology are common features of TBI (Skandsen et al., 2010).

While these preclinical findings are encouraging, heterogeneity in TBI severity, injury location, and patient demographics presents translational challenges that have not yet been addressed in MSC-sEV studies.

### 5.4 Multiple sclerosis

Multiple sclerosis (MS) is a chronic autoimmune disorder affecting approximately 2.8 million people globally, characterized by demyelination, neuroinflammation, and axonal damage in the CNS, leading to progressive neurological disability (Hamidi et al., 2024). Current disease-modifying therapies (DMTs) such as β-interferons, dimethyl fumarate, and anti-CD20 monoclonal antibodies mainly target peripheral immune activation to reduce relapse rates but have limited efficacy in progressive MS and fail to promote CNS repair or remyelination (Hamidi et al., 2024). Furthermore, long-term immunosuppression increases infection risks, while high-dose corticosteroids used for acute relapses can cause metabolic and osteoporotic complications. These unmet clinical needs highlight the urgent requirement for therapies that can combine immunomodulation with neuroprotection and regeneration—a therapeutic profile that MSC-sEVs are uniquely positioned to provide.

The pathophysiology of MS involves autoimmune dysregulation, neuroinflammation, and progressive demyelination, all of which can be targeted by MSC-sEVs (Riazifar et al., 2019). The core of MSC-sEV efficacy in MS lies in immunomodulation. MSC-sEVs suppress pathogenic T-cell proliferation, particularly CD4+CD25– conventional T cells, while enhancing regulatory T cell (Treg) expansion, including CD4+CD25+Foxp3+ populations, thereby restoring immune tolerance (Baharlooi et al., 2021, 2022). This is achieved through dual cytokine regulation: MSC-sEVs downregulate pro-inflammatory mediators (IFN-γ, IL-17, TNF-α, IL-1β, IL-6) and upregulate anti-inflammatory cytokines (IL-10, TGF-β, IL-4), rebalancing the Th17/Treg axis and reducing neuroinflammation in both *in vitro* MS models and experimental autoimmune encephalomyelitis (EAE) mice (Jafarinia et al., 2024).

Preclinical evidence suggests that MSC-sEVs may promote remyelination by delivering miR-23a-3p, which activates the PI3K/Akt pathway and suppresses the Tbr1/Wnt pathway in oligodendrocyte precursor cells (OPCs), promoting their differentiation into mature myelinating oligodendrocytes (Qin et al., 2024). MSC-sEVs enhance myelin basic protein (MBP) expression, reduce demyelinated lesions in spinal cord tissues, and improve neurological function in EAE models (Jafarinia et al., 2024). The oligodendrogenesis-promoting effects are particularly relevant in MS, where remyelination failure is a major contributor to progressive disability (Gugliandolo et al., 2020).

In addition, MSC-sEVs enhance the inhibitory function of Tregs by upregulating lymphocyte-activation gene 3 (Lag-3) in Foxp3+CD4+ T cells, thereby inhibiting immune cell proliferation and cytokine storm in EAE (Riazifar et al., 2019). By modulating microglial polarization toward anti-inflammatory M2 phenotypes, MSC-sEVs resolve chronic inflammation and promote tissue repair (Li et al., 2019). Astrocyte modulation may also contribute to MS therapy by reducing neurotoxic A1 astrocytes that exacerbate demyelination (Liddelow et al., 2017). Unlike traditional DMTs that lack remyelination or neuroprotective effects, MSC-sEVs function as a “multi-drug platform” that can simultaneously inhibit autoimmune attacks, reduce neuroinflammation, and stimulate CNS repair.

Although animal studies support the potential efficacy of MSC-sEV for slowing MS progression, the research data derives primarily from EAE models, which may not fully replicate the complexity of human MS. Clinical applicability of these finding remain to be validated.

## 6 Summary of key MSC-sEVs cargoes and their targets

The multifaceted molecular effects of MSC-sEVs on the CNS described above (Sections 3, 4 and 5) relating to neural protection, neurogenesis, immunomodulation, angiogenesis, and remyelination suggest a coordinated action of diverse bioactive cargo components, possibly including proteins, lipids, and nucleic acids. Among these cargoes, microRNAs (miRNAs) have attracted particular attention as key mediators, owing to their capacity to simultaneously regulate multiple target genes and signaling pathways across different cell types. The principal miRNAs in MSC-sEVs identified as potentially contributing to neural repair and recovery, their molecular targets, downstream pathways, and functional outcomes in the context of neural repair are summarized in Table 1. Notably, individual miRNAs such as the miR-17-92 cluster and miR-21 exert pleiotropic effects across multiple cell types and mechanisms. Furthermore, the collective effects of miRNA cargos can act synergistically within cells to alter their phenotype or coordinate modulation of gene expression in different cells within CNS tissue.

**Table 1:** Key microRNAs in MSC-sEVs: molecular targets, signaling pathways, and functional outcomes in CNS repair.

microRNA	Target genes	Target pathways	Cell types	Biological effect	Section	References
miR-124	REST	Neuronal gene de-repression	Neural progenitor cells (NPCs)	REST suppression leads to de-repression of neuron-specific genes (ion channels/neurotransmitter receptors/synaptic proteins) promoting neuronal differentiation and functional recovery after stroke	3.1	Yang et al., 2017; Conaco et al., 2006
miR-17-92 cluster	PTEN	PI3K/Akt/mTOR	NSCs/OPCs/ neurons	PTEN suppression activates PI3K/Akt/mTOR signaling promoting neurogenesis/oligodendrogenesis/axonal outgrowth/dendritic remodeling/increased dendritic spine density and increased phosphorylation of Akt/mTOR/GSK-3beta	3.1/3.5/4.4/5.1/5.3	Xin et al., 2017
miR-21-5p	PTEN	PTEN/Akt	Cortical neurons	PTEN suppression leads to Akt activation alleviating cortical neuron apoptosis after TBI	3.2	Han et al., 2014
miR-21-5p	PTEN	PTEN/Akt	Neurons	Alleviates early brain injury and improves cognitive function via PTEN/Akt pathway after subarachnoid hemorrhage	3.2	Gao et al., 2020
miR-21-5p	PDCD4	M1/M2 macrophage polarization	Macrophages	PDCD4 suppression shifts macrophage M1/M2 polarization promoting functional recovery after spinal cord injury	3.2/5.1	Li et al., 2025
miR-25	NOX4	Oxidative stress/ROS pathway	Spinal cord neurons	NOX4 inhibition reduces ROS production/decreases malondialdehyde/increases SOD activity protecting neurons from ischemia-reperfusion injury and preserving mitochondrial integrity	3.2/3.4/5.1	Zhao et al., 2019
miR-214-3p	PTEN	PI3K/Akt	Neurons (penumbra zone)	PTEN downregulation restores Akt activation providing neuroprotection and improving neurological function after ischemic stroke	3.3	Wu et al., 2024
miR-133b	RhoA	RhoA/ERK1/2/ STAT3/CREB	Neurons/ astrocytes	RhoA suppression enables cytoskeletal reorganization and growth cone advancement promoting neurite outgrowth and improving hindlimb locomotor recovery after CNS injury	3.5/5.3	Xin et al., 2012; Li et al., 2018
miR-216a-5p	TLR4	TLR4/NF-kappaB	Microglia	TLR4/NF-kappaB suppression shifts microglia from M1 to M2 phenotype in traumatic CNS injury	4.1/7.6.2	Liu et al., 2020
miR-181c	TLR4	TLR4/NF-kappaB (p65)	Microglia/ macrophages	TLR4 downregulation and p65 activation suppression promoting anti-inflammatory phenotype	4.1	Zhang et al., 2021b
miR-146a-5p	TRAF6/NLRP3 inflammasome	NF-kappaB/NLRP3	Macrophages/ microglia	TRAF6 suppression inhibits downstream NF-kappaB signaling promoting M1 to M2 macrophage polarization and inhibiting NLRP3 inflammasome-mediated pyroptosis	4.1	Liang et al., 2024; Hua et al., 2022
miR-125a	IRF5	M1 transcriptional program	Macrophages/ microglia	IRF5 downregulation suppresses master transcription factor driving M1 polarization thereby promoting M2 polarization and neuroprotection	4.1	Chang et al., 2021
miR-873a-5p	NF-kappaB	NF-kappaB	Microglia	NF-kappaB suppression inhibits microglial M1 polarization reducing neuroinflammation (indirect MSC-sEV effect via astrocyte priming)	4.2	Long et al., 2020
miR-126	PIK3R2	PI3K/Akt/VEGF/ Angiopoietin-1	Endothelial cells (HUVECs)	PIK3R2 suppression activates PI3K/Akt signaling promoting endothelial cell proliferation/migration/tube formation and upregulating VEGF and Angiopoietin-1 increasing capillary density	4.3/5.1	Zhang et al., 2021a
miR-210	EFNA3	HIF-1alpha signaling	Endothelial cells	EFNA3 suppression and HIF-1alpha stabilization promoting angiogenesis under hypoxic conditions	4.3/5.1	Moon et al., 2019
miR-23a-3p	Tbr1	PI3K/Akt/Tbr1/ Wnt	Oligodendrocyte precursor cells (OPCs)	PI3K/Akt activation and Tbr1/Wnt pathway suppression promoting OPC differentiation into mature myelinating oligodendrocytes/increasing MBP expression/reducing demyelinated lesions in EAE models	4.4/5.4	Qin et al., 2024

The mechanistic links summarized in this table are derived predominantly from individual preclinical studies, often using engineered overexpression systems. The relative contribution of each miRNA to the overall therapeutic effects of unmodified MSC-sEVs *in vivo* remains to be determined.

Akt, protein kinase B; BDNF, brain-derived neurotrophic factor; CREB, cAMP response element-binding protein; EAE, experimental autoimmune encephalomyelitis; EFNA3, ephrin-A3; ERK1/2, extracellular signal-regulated kinase 1/2; GSK-3β, glycogen synthase kinase-3β; HIF-1α, hypoxia-inducible factor-1α; HUVECs, human umbilical vein endothelial cells; IRF5, interferon regulatory factor 5; MBP, myelin basic protein; mTOR, mammalian target of rapamycin; NF-κB, nuclear factor kappa-light-chain-enhancer of activated B cells; NLRP3, NOD-like receptor protein 3; NOX4, NADPH oxidase 4; NPCs, neural progenitor cells; NSCs, neural stem cells; OPCs, oligodendrocyte precursor cells; PDCD4, programmed cell death protein 4; PI3K, phosphoinositide 3-kinase; PIK3R2, phosphoinositide-3-kinase regulatory subunit 2; PTEN, phosphatase and tensin homolog; REST, RE1-silencing transcription factor; RhoA, Ras homolog family member A; ROS, reactive oxygen species; SOD, superoxide dismutase; STAT3, signal transducer and activator of transcription 3; TBI, traumatic brain injury; TLR4, Toll-like receptor 4; TRAF6, TNF receptor-associated factor 6; VEGF, vascular endothelial growth factor; Wnt, Wingless/Integrated.

## 7 Challenges, future perspectives, and engineering strategies

While MSC-sEVs hold tremendous therapeutic promise for CNS disorders, several challenges must be addressed before widespread clinical translation can be achieved (Gimona et al., 2021).

### 7.1 Standardization, quality control, and storage

A major challenge for clinical translation is the lack of standardized methods for MSC-sEV isolation, characterization, and quality control. Critical quality attributes (CQAs) for MSC-sEV products include particle size distribution, surface marker profiles (CD9, CD63, CD81), zeta potential, protein-to-particle ratio, and cargo composition (miRNA and protein profiles). However, consensus on which CQAs should serve as release criteria for clinical-grade products has not yet been achieved (Gimona et al., 2021). Potency assays present a particular challenge, as functional assays (e.g., immunomodulation, angiogenesis, or neuroprotection assays) may not capture the full spectrum of MSC-sEV actions (Giovannelli et al., 2023). Batch-to-batch comparability remains a significant concern, as isolation method, culture conditions, passage number, and donor variability all influence the composition and potency of the final product (Gimona et al., 2021; Giovannelli et al., 2023).

Regarding product stability, MSC-sEVs can be stored at −80 °C with preserved bioactivity, offering logistical advantages over live cell therapies (Elahi et al., 2020). However, the effects of repeated freeze-thaw cycles on particle integrity, surface marker retention, and cargo bioactivity require systematic characterization for clinical-grade products. Lyophilization has been explored as an alternative storage strategy, but its impact on sEV function in the CNS context remains to be validated (Gimona et al., 2021).

Scalable production technologies, including bioreactor-based culture systems, tangential flow filtration, and size-exclusion chromatography-based isolation, are being developed to meet the demands of clinical-scale Good Manufacturing Practice (GMP) manufacturing. Hollow-fiber bioreactors, in particular, offer continuous sEV production under defined culture conditions, potentially reducing batch-to-batch variability while increasing yield (Gimona et al., 2021).

### 7.2 Biodistribution and pharmacokinetics

Understanding the biodistribution, clearance kinetics, and target tissue accumulation of systemically administered MSC-sEVs is critical for dose optimization and safety assessment. Route-dependent biodistribution is a critical determinant of therapeutic efficacy. While MSC-sEVs delivered by the IV, and IN route both reach the CNS within 24 hours of administration in a mouse model, the fraction of sEVs reaching the brain and spinal cord and the speed of delivery to the CNS vary with the route of administration (Tolomeo et al., 2023). Observed therapeutic efficacy for SCI and other CNS disorders using IV, IT, and IN delivery argue that all 3 routes of administration may be feasible (Tolomeo et al., 2023). However, each method has different advantages and limitations. IV delivery is highly compatible with standard hospital procedures but reaches the CNS more slowly and at lower concentrations than IN or IT delivery. IN delivery offers a rapid route of delivery to the cerebral spinal fluid and ease of patient self-administration by patients but requires training for consistent dosing. IT administration rapidly and consistently administers treatment into the CNS but requires a trained neurosurgeon to deliver and presents a challenge for repeated dosing. Dosing decisions for MSC-sEVs are complicated by the lack of standardized quantification methods across studies; particle number, total protein content, and specific marker concentrations are all used, but none alone adequately predicts therapeutic potency (Gimona et al., 2021).

### 7.3 Safety and regulatory considerations

MSC-sEVs have low tumorigenic risk compared to cell-based therapies, but do not fit existing drug or cell therapy regulatory categories, complicating approval pathways and GMP compliance (Gimona et al., 2021). Data on long-term immunogenicity and repeated dosing remain limited (Gimona et al., 2021). Regarding immune responses, MSC-sEVs are generally considered to have a favorable safety profile, with lower immunogenicity and no risk of autonomous replication compared to their parent cells (Gimona et al., 2021; Lotfy et al., 2023). Nevertheless, several aspects warrant further investigation for clinical translation. The immunological consequences of repeated MSC-sEV administration have not been systematically evaluated, particularly in the CNS context (Gimona et al., 2021; Giovannelli et al., 2023). Notably, the apparent inhibition of inflammatory processes by MSC-sEVs could increase infection risks in some circumstances. Regarding tumor-promoting potential, although MSC-sEVs lack the capacity for autonomous replication, their pro-angiogenic and anti-apoptotic cargo could theoretically interact with pre-existing malignancies; however, no increased tumor incidence has been reported in preclinical studies to date (Giovannelli et al., 2023). Long-term safety data remain limited, as most preclinical studies assess outcomes over weeks to months; the cumulative safety profile for chronic conditions requiring sustained treatment will need to be established through extended clinical follow-up (Elahi et al., 2020).

Notably, a recent systematic review identified 66 registered clinical trials evaluating MSC-derived EVs across diverse indications between 2014 and 2024 (Wang et al., 2025), with conditions including COVID-19 ARDS, Crohn's disease, osteoarthritis, chronic kidney disease, and graft-versus-host disease. Among these, a phase 2 randomized, placebo-controlled dosing study of BM-MSC-derived EVs for COVID-19 respiratory failure demonstrated safety with no treatment-related serious adverse events (Lightner et al., 2023), an early-phase study in chronic kidney disease showed improvements in estimated glomerular filtration rate and anti-inflammatory markers (Nassar et al., 2016), and a case report documented clinical benefit in therapy-refractory graft-versus-host disease (Kordelas et al., 2014). Across these trials, MSC-sEV therapies have demonstrated a favorable safety profile with low rates of serious adverse events, providing advantages over MSC cellular injection by reducing infusion-related toxicities (Lotfy et al., 2023). Intravenous infusion and aerosolized inhalation have emerged as the predominant administration routes, with nebulization achieving therapeutic effects at lower particle doses than intravenous delivery, suggesting route-dependent dose optimization will be critical (Wang et al., 2025). The safety and feasibility data emerging from these trials will be informative for the design of future CNS-focused studies.

To date, no completed clinical trials have specifically evaluated MSC-sEV therapy for neurological disorders. However, as the number of non-neurological MSC-sEV trials continues to grow, the safety profile of MSC-sEV administration in humans is becoming increasingly well characterized, which should directly inform and accelerate the design of CNS-focused studies. Such trials will need to address dose selection, route of administration, and patient stratification based on disease stage and severity. The optimal dose, frequency, route, and duration of MSC-sEV treatment will need to be determined for each indication, and these parameters may interact in complex ways.

### 7.4 Therapeutic window

The therapeutic window depends on both the condition and the mechanism being targeted. Acute neuroprotection requires early intervention, while immunomodulation, vascular repair, and remyelination-promoting effects may be achievable with delayed administration. In rodent models of stroke, MSC-sEVs have shown efficacy when administered at 24 h post-injury (Xin et al., 2013a) and even in the chronic phase (Xia et al., 2020). In spinal cord injury, MSC-sEV treatment initiated at 7 days post-injury improved functional recovery (Nakazaki et al., 2021), and infusion of the parent MSCs has demonstrated efficacy when treatment was delayed up to 10 weeks post-SCI (Morita et al., 2016). If IV-infused MSCs in the chronic setting act via release of sEVs, as proposed for subacute treatment, it is likely that direct MSC-sEV administration would also be effective in the chronic phase. For neurodegenerative conditions such as Alzheimer's disease, the chronic progressive nature of the pathology may require sustained or repeated treatment over months to years.

### 7.5 Dosing strategies

Dosing strategies represent another critical variable. Our previous work demonstrated that fractionated dosing of MSC-sEVs over three consecutive days enhanced BSCB recovery compared to single-dose administration with the same total quantity of sEVs (Nakazaki et al., 2021), suggesting that sustained exposure may amplify therapeutic effects. This is consistent with the observation that IV-infused MSCs lodge in the lungs and release sEVs over approximately 2–3 days (Matsushita et al., 2015), providing a natural model of fractionated delivery. More recently, we demonstrated that continuous intravenous infusion of hMSC-sEVs via osmotic pump over three days accelerated the onset of motor recovery compared to daily injections, and extending the infusion to six days further enhanced recovery despite the same total dose (Nakazaki et al., 2026a). Molecular analyses revealed that hMSC-sEVs suppressed macrophage-mediated extracellular matrix deposition at the lesion site, suggesting that prolonged continuous delivery optimizes the interaction between sEVs and target cells at the injury site. Dosages of MSC-sEVs reported to improve functional outcomes in rodent SCI models have varied widely, and the lack of clear correlation between total dosage and magnitude of recovery across studies suggests that other parameters—including timing, route of delivery, and fractionation schedule—may be more important determinants of efficacy than absolute dose alone (Nakazaki et al., 2021).

### 7.6 Engineering strategies for enhanced efficacy

Several engineering approaches are being developed to enhance MSC-sEV therapeutic efficacy:

#### 7.6.1 Surface modification for targeted delivery

Delivery route affects biodistribution and efficacy. Intravenous administration is practical and has shown efficacy in preclinical studies, but brain targeting may be enhanced through surface modification. Cui et al. (2019) demonstrated that RVG-modified MSC-sEVs showed improved targeting to the cortex and hippocampus with superior therapeutic effects in AD mice (Cui et al., 2019). Moreover, several surface engineering approaches are being explored for CNS-targeted delivery (Yang et al., 2025), including click chemistry-based conjugation of targeting ligands (Yang et al., 2025), magnetic nanoparticle loading for magnetically guided delivery (Jiang et al., 2025), and genetic engineering and preprocessing of source cells to enhance targeting and yield (Liao et al., 2025). However, surface modifications may alter sEV biodistribution, clearance kinetics, and cargo bioactivity, and these parameters require systematic optimization for each targeting strategy.

#### 7.6.2 Preconditioning strategies

Source cell preparation is critical for therapeutic potency. Hypoxia preconditioning of MSCs enhances the therapeutic potency of derived sEVs by increasing the content of neuroprotective miRNAs (Liu et al., 2020). Exosomal miR-216a-5p derived from hypoxia-preconditioned BM-MSCs shifts microglia from M1 to M2 via TLR4/NF-κB suppression (Liu et al., 2020). Similarly, IL-4 priming of MSCs enhances the anti-inflammatory capacity of derived sEVs through enrichment of miR-21-5p (Li et al., 2025). These preconditioning strategies offer a practical approach to enhance therapeutic efficacy without genetic modification (Liu et al., 2020; Li et al., 2025).

#### 7.6.3 Cargo engineering

In addition to miRNAs and proteins, emerging evidence implicates other non-coding RNAs in MSC-sEV-mediated effects. Long non-coding RNA (lncRNA)-Gm37494-loaded exosomes from hypoxia-preconditioned adipose tissue-derived MSCs have been shown to regulate M1/M2 polarization of microglia through the miR-130b-3p/PPARγ axis, demonstrating enhanced therapeutic efficacy in spinal cord injury (Shao et al., 2020). Given that endogenous circular RNAs (circRNAs) such as CDR1as regulate fibrosis through the miR-7a-5p/TGF-βR2/Smad pathway in spinal cord injury (Wang et al., 2024), engineering MSC-sEVs to deliver functional lncRNAs and circRNAs, in addition to miRNAs and proteins, represents a promising avenue for cargo optimization.

Beyond nucleic acid cargo, sEVs can also be loaded with small-molecule therapeutics. Curcumin-laden exosomes have been shown to target ischemic brain tissue and alleviate cerebral ischemia-reperfusion injury by inhibiting ROS-mediated mitochondrial apoptosis (He et al., 2020), and curcumin-primed UC-MSC-derived EVs improved motor recovery in a complete spinal cord injury model by reducing inflammation and enhancing axonal regeneration (Xiong et al., 2023), demonstrating the feasibility of pharmacological cargo loading of MSC-sEVs for CNS applications.

#### 7.6.4 Scaffold-based sustained release systems

Systemic MSC-sEV administration suffers from rapid clearance and limited accumulation at injury sites (Lankford et al., 2018). Biomaterial-based delivery systems address this limitation by enabling localized, sustained release directly at the lesion site while providing structural support for tissue regeneration (Liao et al., 2025). Hajinejad et al. (2023) demonstrated that combining human neural stem cell-derived exosomes with a nano-scaffold containing SDF-1α (Nano-SDF) enhanced therapeutic outcomes in TBI, reducing oxidative stress and neuroinflammation while promoting neurogenesis through a “bio-bridge” mechanism—an approach that may be translatable to MSC-sEV-based therapies (Hajinejad et al., 2023). Similarly, Deng et al. (2025) showed that decellularized tissue matrix hydrogels functionalized with EVs promote macrophage reprogramming and neural stem cell differentiation in spinal cord injury (Deng et al., 2025). Injectable hydrogels with tunable release kinetics and stimulus-responsive systems represent promising approaches for optimizing spatiotemporal control of sEV delivery to CNS lesions (Liao et al., 2025).

#### 7.6.5 Synthetic and biomimetic EV platforms

Synthetic nanoparticles engineered to mimic the surface properties and cargo delivery functions of natural sEVs represent an emerging approach that may overcome batch variability and scalability challenges inherent to cell-derived products (Jiang et al., 2025; Yang et al., 2025). Biomimetic vesicles, including cell membrane-coated nanoparticles and engineered liposomes loaded with therapeutic miRNAs, can potentially recapitulate the targeting and immunomodulatory properties of MSC-sEVs while enabling standardized, large-scale production (Jiang et al., 2025; Yang et al., 2025). However, these platforms are in early stages of development, and whether they can fully replicate the complex, multifactorial cargo of natural MSC-sEVs remains to be determined (Lotfy et al., 2023).

### 7.7 Improvement of translational models

Current preclinical evidence for MSC-sEVs predominantly derives from rodent models, which provide valuable mechanistic insights but may not fully recapitulate human CNS pathophysiology (Alizadeh et al., 2019). Large animal models including porcine and non-human primate models offer closer anatomical and physiological parallels to humans, particularly regarding CNS dimensions, CSF dynamics, and immune responses (Williams et al., 2020; Go et al., 2021). However, the extensive clinical safety and efficacy data already accumulated for the parent MSCs in stroke (Honmou et al., 2011; Hassanein et al., 2023), spinal cord injury (Honmou et al., 2021), and Alzheimer's disease (Rash et al., 2025) may help to de-risk the translational pathway for MSC-sEVs; however, it should not be assumed that MSC-sEV products will simply replicate the effects of whole-cell therapy, as the two approaches may differ in biodistribution, pharmacokinetics, and the spectrum of bioactive factors delivered. Organoid-based *in vitro* systems and patient-derived iPSC models represent complementary approaches for mechanistic studies and personalized medicine applications (Lee et al., 2022).

### 7.8 Future directions

Advancing MSC-sEVs toward clinical application will require progress on multiple fronts simultaneously. Transitioning from laboratory-scale isolation to GMP-compliant manufacturing pipelines with defined culture media, reproducible isolation techniques, and robust potency assays will be essential for producing a consistent, well-characterized treatment product (Gimona et al., 2021). The rapid clearance and off-target accumulation of systemically administered sEVs motivate continued development of surface modification for CNS targeting and scaffold-based sustained release, as well as combination approaches that pair MSC-sEVs with biomaterial scaffolds, electrical stimulation, or rehabilitative training (Liao et al., 2025). Optimal dose, fractionation schedule, route, and timing of administration will need to be established for each indication through systematic preclinical and clinical studies. The extensive clinical safety and efficacy data already accumulated for parent MSCs provide a foundation that may substantially de-risk and expedite the path to human trials for MSC-sEVs, and the growing body of evidence from MSC-sEV trials in non-neurological conditions will further inform the design of CNS-focused studies. The pace of clinical translation will ultimately depend on the convergence of standardized production, optimized delivery, and rigorous clinical validation.

## 8 Conclusion

The therapeutic effects of intravenously administered MSCs in CNS disorders appear to be mediated largely by small extracellular vesicles released from MSCs lodged in the pulmonary vasculature. These MSC-sEVs traffic to sites of CNS injury and exert their effects through two convergent categories of action. Directly, MSC-sEVs promote neuronal survival via miR-21 and miR-25-mediated anti-apoptotic and antioxidant pathways, preserve mitochondrial function through PINK1/Parkin-mediated mitophagy, stimulate endogenous neural stem cell activation via Wnt/β-catenin signaling and miR-124-driven suppression of REST, and enhance neurite outgrowth and synaptogenesis through miR-133b and the miR-17-92 cluster. Indirectly, MSC-sEVs reshape the injury microenvironment by shifting microglia/macrophages toward anti-inflammatory phenotypes, converting neurotoxic astrocytes to neuroprotective states, restoring vascular integrity and promoting angiogenesis, and facilitating oligodendrogenesis and remyelination—with particular relevance to protecting TNF-α-sensitive oligodendrocyte precursor cells.

These mechanisms collectively address key features of the pathophysiology of stroke, Alzheimer's disease, vascular dementia, traumatic brain injury, and multiple sclerosis, and preclinical evidence across these conditions is substantial and growing. Clinical translation will require standardized GMP-compliant manufacturing of a consistent treatment product, optimization of dose, timing, and route of delivery for each indication, and resolution of regulatory pathways for this novel class of cell-free biologics. The extensive clinical experience already accumulated with the parent MSCs, together with early-phase MSC-sEV trials underway for non-neurological conditions, provides a foundation that may substantially de-risk and accelerate this translational pathway. MSC-sEVs hold considerable potential as a cell-free therapeutic approach for neurological disorders that currently lack effective regenerative treatments, although the translation of preclinical promise to clinical application will require rigorous validation specific to MSC-sEV products, rather than reliance on clinical data from parent MSC therapies alone.
